# Development of machine learning models to predict clinical outcome and recovery time in dogs with parvovirus enteritis

**DOI:** 10.3389/fvets.2025.1555714

**Published:** 2025-04-15

**Authors:** Negin Sanaei, Mohamad Zamani-Ahmadmahmudi, Seyed Mahdi Nassiri

**Affiliations:** ^1^Department of Clinical Pathology, Faculty of Veterinary Medicine, University of Tehran, Tehran, Iran; ^2^Department of Clinical Science, Faculty of Veterinary Medicine, Shahid Bahonar University of Kerman, Kerman, Iran

**Keywords:** canine parvovirus, machine learning, survival, prediction, dog

## Abstract

Canine parvovirus (CPV) is one of the most contagious viral diseases in dogs that usually presents with diarrhea, vomiting, and fever. Various clinical and laboratory biomarkers such as SIRS, leukopenia, neutropenia and CRP have been introduced to predict the final outcome of dogs with CPV. With the advent of machine learning methods/algorithms, various models can be developed using a combination of clinical and non-clinical variables to predict clinical outcome in different diseases with higher efficiency compared to traditional biomarkers. In this study, we sought to develop models to predict clinical outcome and recovery time in dogs with CPV infection using 10 and 4 machine learning algorithms, respectively. A model was developed using four variables (SIRS, deworming, vaccination and crying) to predict clinical outcome. The performance of this model was measured using three metrics: accuracy scores, AUC (area under the Receiver Operating Characteristic (ROC) curve) and AUC score. Another model was constructed using five variables (retching, foul smelling, housing, dehydration, and shift-to-left) to estimate recovery time. The performance of this model was evaluated using two criteria: mean square error (MSE) and root mean square error (RMSE). In the model developed for clinical outcome, the average of accuracy scores, AUC scores and AUCs in the test dataset were 0.84, 0.90 and 0.73, respectively. The second model predicted the recovery time in the test group with a mean error of 2 days (RMSE = 2.05). Our findings demonstrate that ML models can effectively integrate clinical and laboratory features to predict survival and recovery time in CPV-infected dogs, offering a valuable tool for early prognosis and treatment optimization.

## Introduction

Canine parvovirus type 2 (CPV-2), which belongs to the genus Protoparvovirus in the family Parvoviridae, is a single-stranded DNA virus that is ~5.12 kb in length (1). CPV spreads rapidly in the canine population and has a high mortality rate. Because CPV is completely dependent on the host cell, virus replication requires cells with high proliferative capacity, such as the digestive tract, bone marrow, and lymphoid tissues. CPV infection primarily affects three main tissues: the GI tract, bone marrow, and myocardium, though the skin and nervous tissue may also be impacted. The most common clinical manifestations are diarrhea, vomiting, and fever. The severity of vomiting is often severe, and diarrhea and anorexia occur with less severity. Excretion of body fluids and proteins through the digestive system causes severe dehydration and hypovolemic shock (2–5).

One of the most important aspects of CPV for the small animal practitioner and animal owner is the prediction of clinical outcome in infected dogs. Therefore, various clinical and laboratory biomarkers have been introduced to estimate the prognosis in dogs with CPV. For example, some studies have shown that leukopenia, neutropenia, and lymphopenia as important biomarkers for predicting clinical outcome, while other studies have shown that SIRS syndrome is a strong risk factor for non-survival patients (5–8). In addition, it was reported that serum levels of C-reactive protein and ceruloplasmin were significantly higher in non-survival dogs than in survival dogs (9). Serum cortisol, thyroxine concentrations and C-reactive protein (CRP) have also been reported as other prognostic biomarkers in canine parvovirus enteritis (10, 11). Although all these biomarkers are individually valuable prognostic factors, their combination can create stronger and more reliable prognostic indicators. The main disadvantage of these single biomarkers is the lack of reproducibility, as many times a biomarker is confirmed in one study while not in another. The difficulty of measurement and cost are other major disadvantages. It would be a great advantage if we could build prognostic models using variables that can be recorded/measured easily and with minimal cost/effort. Recent advances in machine learning (ML) and artificial intelligence (AI) have provided us with various valuable algorithms that are widely used to predict the clinical outcome of various diseases in human and animals using a combination of multiple variables rather than based on a single variable (12–17). Furthermore, attempts are made to use variables that are easily measurable to enter ML models in most such studies. Although the use of ML to study human diseases in various aspects is relatively widespread, the application of these technologies in veterinary medicine and especially small animal medicine is in its early stages (18). Predicting clinical outcome (survival) and recovery time is very important for small animal physicians and dog owners. While single prognostic biomarkers are not enough powerful predictors, developing ML models using multiple clinical/non-clinical variables can more robustly and reliably predict outcome and recovery time. For example using *random forest* algorithm and some of the hematology and serum biochemistry variables including antithrombin, serum aspartate aminotransferase, serum lipase, monocyte and lymphocyte count, the survival time could be reliably predicted (18). Given the lack of predictive models in CPV, this study aimed to develop an ML-based model to predict clinical outcome and recovery time in 156 dogs with CPV.

## Methods

### Patients

Current study was done on 156 dogs with confirmed CPV infection referred to the University of Tehran of Veterinary Medicine Hospital or private clinics. In addition to clinical/historical symptoms (depression, diarrhea, vomiting), CPV infection was confirmed using a rapid fecal antigen test (Arvin Biohealth: Iran, specificity: 100%, sensitivity: 97.6%). As recommended by the kit manufacturer (Arvin Biohealth: Iran), in case of vaccination, at least 10 days must have passed since the vaccination to consider the test positive. Dogs with confirmed CPV test and complete laboratory, clinical, and outcome variables were included in the study. Dogs were excluded from the study if they did not have any of the hematology, biochemistry, or clinical examination parameters or outcome (see “Data collection” section for details on recording variables). There were no inclusion/exclusion criteria based on age, gender, and severity of disease.

### Data collection

For each CPV-infected case, we collected three types of data: (1) demographic, (2) clinical, and (3) laboratory variables, all recorded prior to treatment initiation (Table 1). This study aims to develop an ML model to predict clinical outcomes and recovery time in CPV-infected dogs using easily obtainable demographic, clinical, and laboratory variables. Regarding laboratory variables, we only included hematology variables (e.g., WBC, neutrophil count, and left shift) and some biochemical analytes (glucose, magnesium, and paraoxonase) that were confirmed in previous studies as prognostic parameters for CPV (5, 6, 19–22).

**Table 1:** Various type of data (variables) recorded for each dog with CPV.

**Demographic variables**	**Clinical variables**	**Laboratory variables**
Age	Clinical signs^a^	WBC(/μl)
Gender	Temperature^b^	RBC (/μl)
Breed	Fever^b^	HGB (g/dl)
Housing	Time of anorexia	HCT (%)
Vaccination	Fecal antigen test	PLT (/μl)
Deworming	Anorexia	LY (/μl)
Vaccination of mother	Lethargia	MO (/μl)
History of stress	SIRS	EO (/μl)
	Vomiting	GR (/μl)
	Diarrhea	Band (/μl)
	Foul smelling	RDWCV
	Dyspnea	Neutrophil/lymphocyte ratio (NLR)
	Crying	Platelet/lymphocyte ratio (PLR)
	Retching	Leukopenia
	Lymphadenomegaly	Neutropenia
	Pale mucous	Lymphopenia
	Dehydration	Shift to left
	Heart rate	Mg (mg/dl)
	Respiratory rate	Glucose (mg/dl)
	Abnormal heart sound	Paraxonase (PON) (U/ml)
	Abnormal respiratory sound	
	Outcome (survival/none-survival)	
	Time of recovery	

^a^Clinical signs mean signs suggestive of CPV infection.

^b^Temperature is body temperature as a continuous variable while fever is a categorical variable (yes or no).

First, a questionnaire was designed to record demographic information and clinical metadata. In the questionnaire, demographic information (such as age, sex, breed, housing, nutrition, vaccination, and antiparasitic treatment), clinical symptoms and the presence of systemic inflammatory response syndrome (SIRS) were recorded. SIRS was confirmed by the presence of at least three of the four criteria, including heart rate > 140/min, respiratory rate > 30/min, body temperature > 39.2°C, and total white blood cell count more than 17,000/μl or < 6,000/μl (5). Recovery time was considered as the interval (days) between confirmation of CPV infection and complete clinical recovery. Afterwards, whole blood was sampled for routine complete blood count (CBC) test and measurement of previously confirmed serum biomarkers for canine CPV infection (glucose, magnesium, and paraoxonase) (21, 22) (Table 1). The samples were taken with the consent of the animal owner. CBC test was performed using a veterinary hematology analyzer (Celltacα, NIHON KHODEN, Japan) and glucose, Mg, and PON were measured using colorimetric GOD/PAP, colorimetric Xylidyl Blue, and colorimetric sandwich ELISA kit (ZellBio GmbH, Germany), respectively. Also, for 47 cases, a second CBC test after treatments (postadmission sampling) was performed and relevant data was recorded.

### Primary data analysis

Before developing ML models to predict clinical outcome or estimate recovery time, we performed a preliminary statistical analysis on various clinical/none-clinical data. Descriptive analysis was performed on various categorical and numerical variables using *SPSS 23* software (Chicago, IL, USA). Also, the relationship between clinical outcome and numerical variables or clinical outcome and categorical variables was done using one-way ANOVA and Pearson's chi-squared test, respectively. *P* < 0.05 level was considered significant.

### Developing ML model to predict clinical outcome

**Figure 1:**
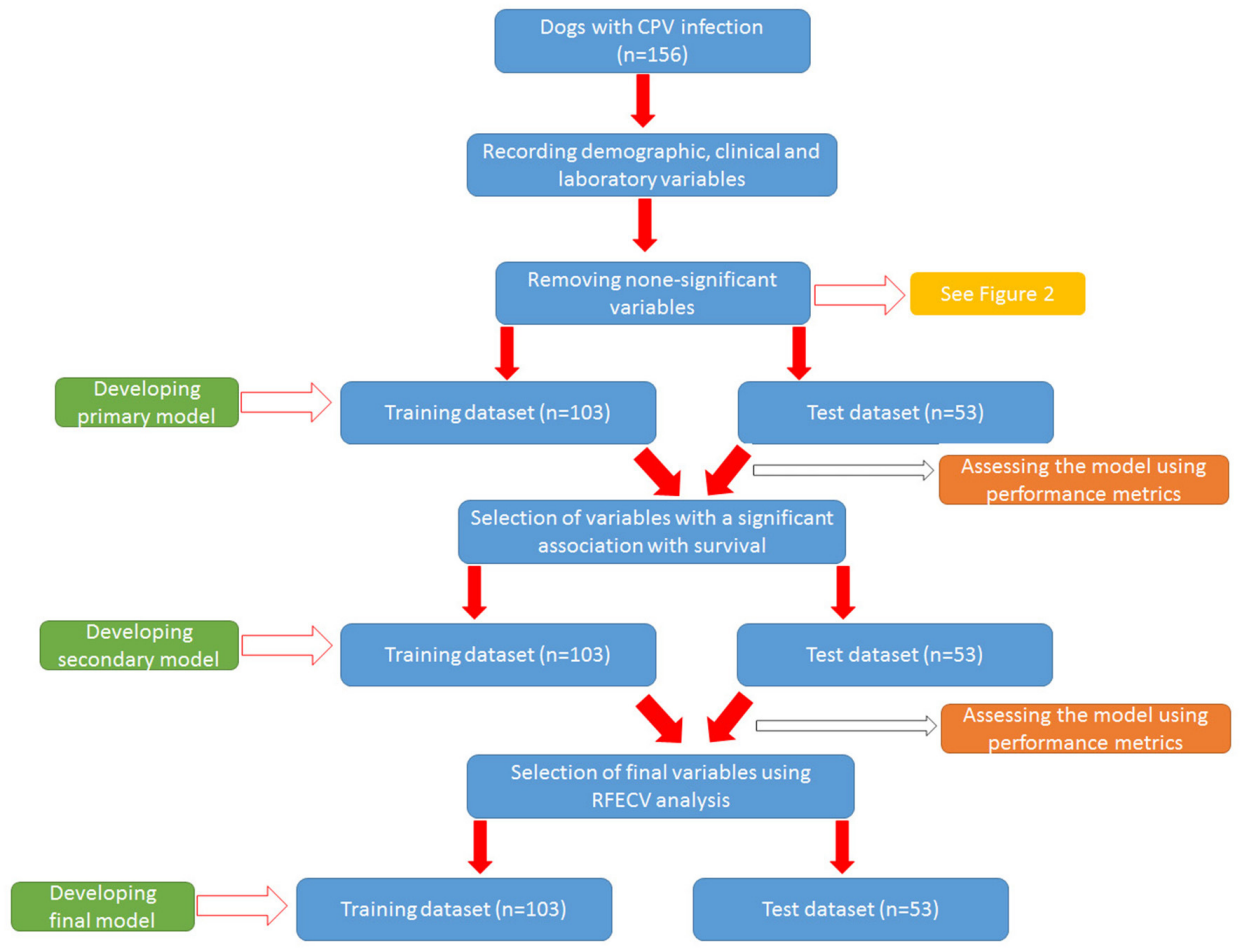
The general workflow for developing models to predict clinical outcome.

The general workflow for developing models to predict clinical outcome is summarized in Figure 1.

#### Filtering the variables

Before developing the model, we revised the initial variables to filter out unnecessary variables. Our workflow for removing non-informative variables is summarized in Supplementary Figure 1. Briefly, variables with more than 25% missing data, variables with small changes (low information), duplicate variables and dummy variables with more than 10 levels were identified and removed. Dummy variables can have two or more levels. A variable with small change is a variable in which 90% of the samples have the same information. For example, more than 90% of cases in our study had anorexia. Duplicate variables are continuous or categorical variables that provide the same information (such as temperature and fever) (Supplementary Figure 1).

There were few missing data for some of the variables. In this situation, the missing values for continuous and categorical data were filled with the average value and the value with the highest frequency, respectively.

#### Training the outcome models

**Figure 2:**
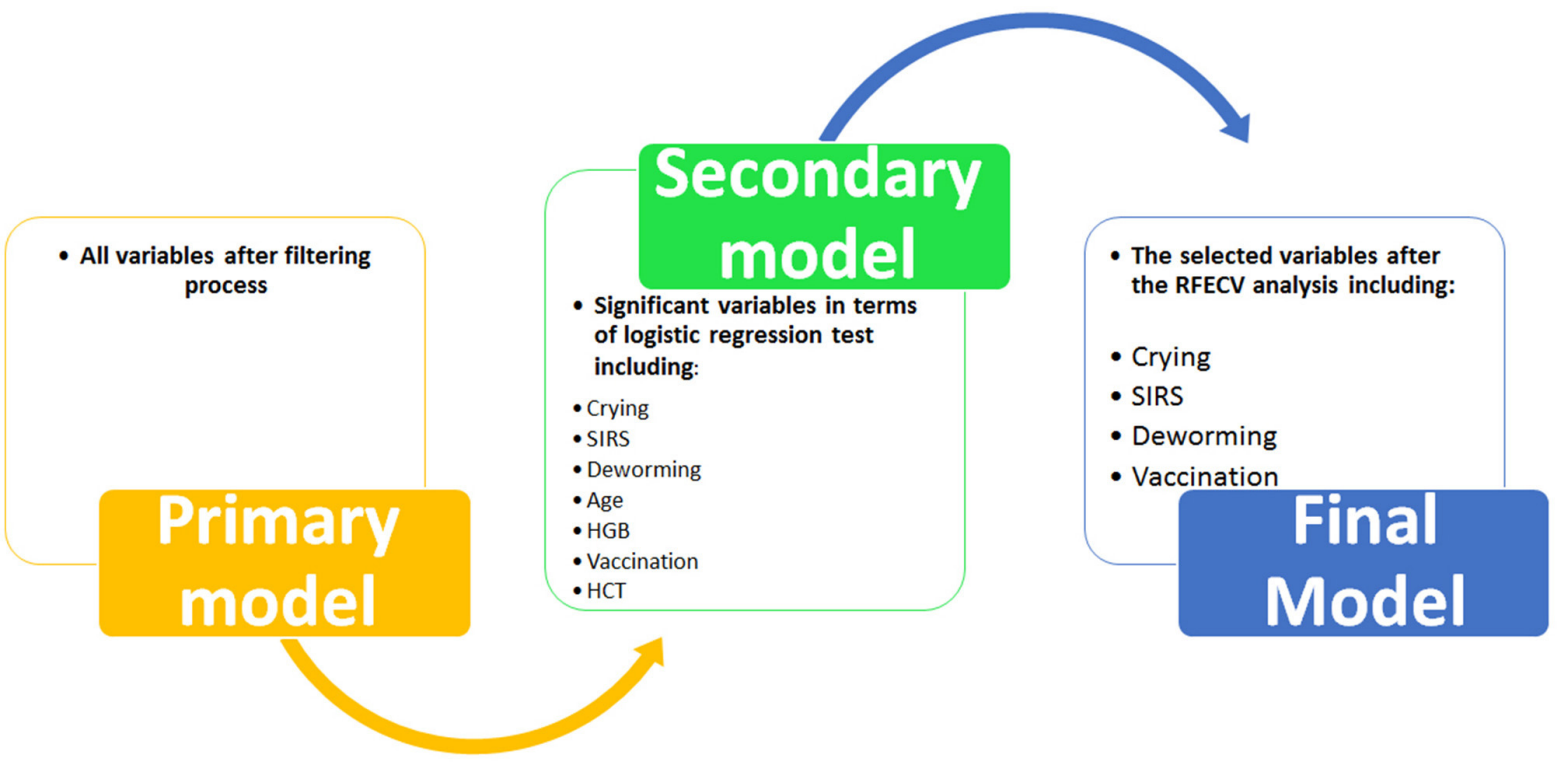
Development process of different models to predict clinical outcome.

After removing non-informative variables, different ML models were trained to reach a final optimal model. Figure 2 shows the workflow for developing the final model. To develop each model, the initial dataset was first divided into training dataset (67%, *n* = 104) and test (validation) dataset (33%, *n* = 51). Then the model was built in the training dataset using different algorithms and then used to predict the clinical outcome in the test dataset. The algorithms used to build the models were from the *scikit-learn* library and include *LogisticRegression, Support Vector Classification (SVC), GaussianProcessClassifier, DecisionTreeClassifier, RandomForestClassifier, AdaBoostClassifier, Gaussian Naive Bayes (GaussianNB), QuadraticDiscriminantAnalysis, LinearDiscriminantAnalysis*, and *GradientBoostingClassifier*. The performance of the models was evaluated using three parameters: accuracy score, AUC [Area under the Receiver Operating Characteristic (ROC) Curve], and AUC score (23). Accuracy score simply indicated a percentage of correct predictions made by a model. The AUC indicates how well the model can discriminate the classes, while the AUC score indicates how reliable the AUC value is. As a rule of thumb, AUCs between 0.6 and 0.7 show poor discrimination, AUCs between 0.7 and 0.8 indicate acceptable discrimination and AUCs between 0.8 and 0.9 indicate excellent discrimination. To tackle the imbalance problem in our analysis, we also trained and compared models with and without SMOTE (Synthetic Minority Over-sampling Technique) analysis. We merely used SMOTE analysis in the training group. To control the problem of overfitting during model development, K-fold cross validation (https://scikitlearn.org/stable/modules/cross_validation.html) was performed in both training and test groups for all algorithms. In our analysis, we considered *k* = 10 in the cross-validation analysis. All models were built using python language and Jupyter notebook. Additionally, we tuned our models to find optimal values of the hyperparamters using the *GridSearchCV* tool in the scikit-learn library.

First, a primary model was trained using all the variables selected in the previous step. As the primary model required too many variables and showed poor performance (see Results section), a secondary model was constructed. To reduce the number of primary variables, logistic regression analysis was performed to identify variables with a significant association with the outcome (survival) (*Ps* < 0.05). Then the variables that had a significant relationship with the outcome were included in the process of building the secondary model. Next, the secondary model was evaluated using performance parameters. Although the performance of the secondary model was significantly better than the primary model (see Results section), the AUCs (as the most important performance metric) of this model were not ideal. Hence we developed the third model (i.e., the final model) with the help of Recursive Feature Elimination with Cross-Validation Analysis (RFECV) to select considerable features (variables) (24). In this analysis, the number of features (variable) selected is tuned automatically by fitting an RFE selector on the different cross- validation splits. As a result, selected and non-selected variables were labeled as True and False, respectively. Using RFECV analysis, four variables (crying, SIRS, deworming and vaccination) were selected and included in the final model (Figure 2). Again, the performance of the final model was checked in both training and test groups using the mentioned parameters.

### Developing ML model to predict recovery time

#### Filtering the variables

We also attempted to develop an optimized model(s) that robustly predicted the time to recovery in studied dogs with CPV infection. The initial stage for these models was similar to the models developed for clinical outcome. Therefore, we first filtered the unnecessary variables using the workflow mentioned above (Supplementary Figure 1).

#### Training the recovery models

**Figure 3:**
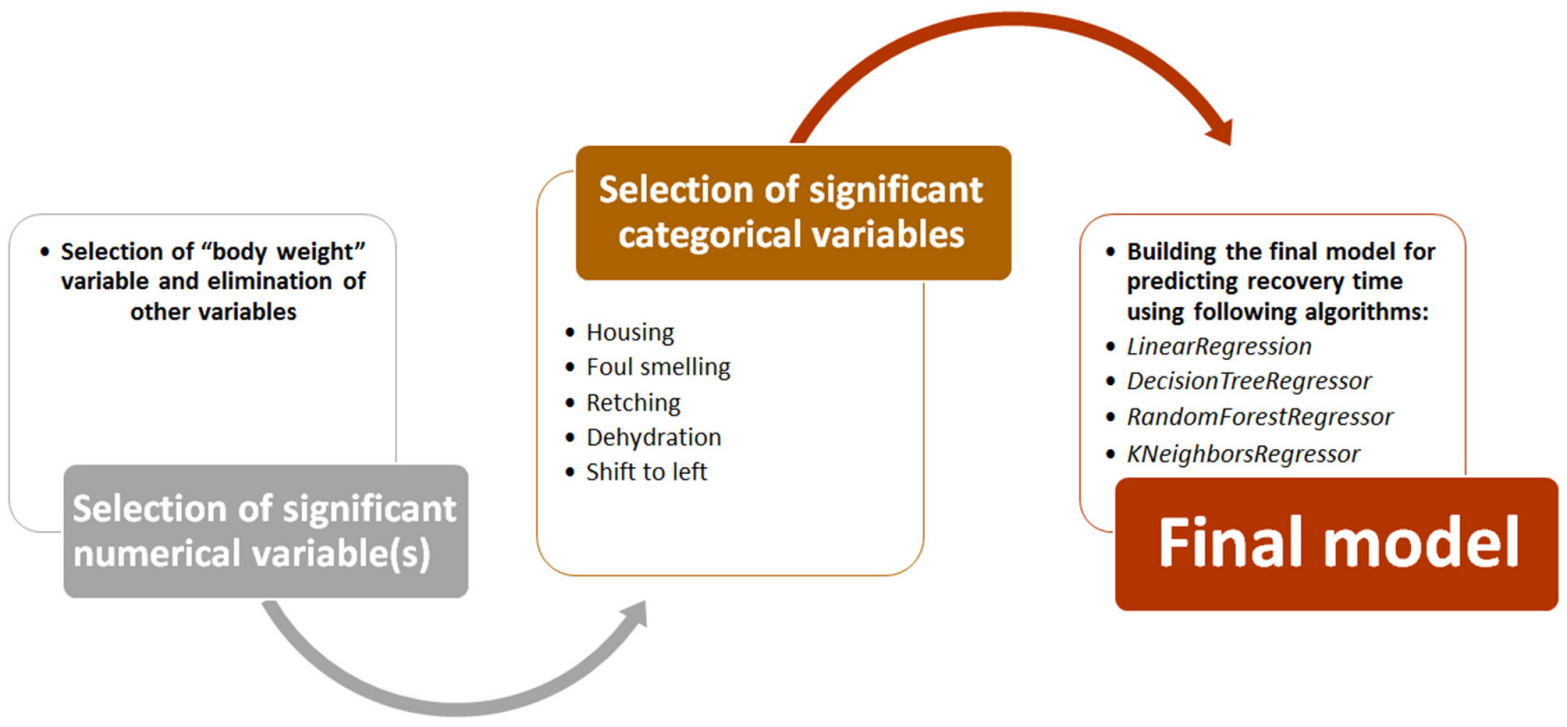
Development process of different models to predict recovery time.

To build the final predictive model, we followed the path shown in Figure 3. First, numerical variables (e.g., age and CBC data) that had a significant correlation with recovery time were identified using Spearman's correlation analysis. Then, categorical variables (e.g., deworming, SIRS, housing, dehydration, and foul smelling) with a significant relationship with recovery time were identified using ANOVA analysis. Both analyzes were performed using *Pandas* and *statsmodels* libraries in Jupyter notebook. Finally, using significant numerical and categorical variables, ML models were developed using four regression algorithms from *scikit-learn* library (*LinearRegression, DecisionTreeRegressor, RandomForestRegressor*, and *KNeighborsRegressor*). Here again, the models were trained using 67% of the dataset and tested on the remaining 33% of the data. The performance of these models was evaluated based on the mean square error (MSE) and root mean square error (RMSE) parameters (25). Both metrics represent the average difference between estimated and actual values. However, since RMASES are the root value of MASES, they provide more realistic and tangible differences.

## Results

### Descriptive analysis of the studied cases

In our study, we worked on 156 dogs with CPV infection with average body weight of 7.96 ± 6.28 kg and average age of 4.64 ± 4.0 months. German shepherded (28.6%), mix dogs (23.4%), Pomeranian (9.1%), and Sarabi (7.8%) were most common breeds in our study. Table 2 shows the frequency of demographic variables. As shown, the frequency of vaccination and deworming variables was significantly different between the survival and none-survival groups. The frequency of vaccinated and dewormed dogs in the survival group was significantly higher than the non-survival group (*Ps* < 0.05). Also, the frequency of the gender variable tended to be significant (*P* = 0.057) (Table 2). Frequency of clinical categorical and clinical numerical variables are presented in Tables 3, 4, respectively. Here we found that the number of cases with SIRS, dyspnea, and crying in the non-survival group was significantly higher than the survival group (*Ps* < 0.05) (Table 3).

**Table 2:** Frequency of demographic variables in studied dogs with CPV.

		**None-survivor**	**Survivor**	**Total**	***P*-value**
Gender	Male	22	65	87	0.057
	Female	8	55	63	
Housing^a^	No	12	56	68	0.512
	Yes	18	64	82	
Vaccination	No	21	57	78	**0.027**
	Yes	9	63	72	
Deworming^b^	No	21	58	79	**0.034**
	Yes	9	62	71	
Vaccination of mother	No	12	49	61	0.446
	Yes	16	47	63	

The frequency of each variable has been compared between survivor and non-survivor groups.

^a^Case was indoor (yes) or outdoor (no).

^b^Deworming means recent deworming. The bold values indicate statistically significant differences.

**Table 3:** Frequency of clinical categorical variables in studied dogs with CPV.

		**None-survivor**	**Survivor**	***P*-value**
History of stress^a^	Negative	21	86	0.857
	Positive	9	34	
Fever	Negative	16	86	0.076
	Positive	13	34	
CPV kit	Negative	1	3	0.800
	Positive	29	117	
Anorexia	Negative	2	4	0.405
	Positive	28	116	
Lethargy	Negative	3	20	0.365
	Positive	27	100	
SIRS	Negative	15	91	**0.005**
	Positive	15	29	
Vomiting	Negative	7	20	0.395
	Positive	23	100	
Diarrhea	Negative	3	12	0.989
	Positive	27	107	
Foul smelling	Negative	1	16	0.122
	Positive	29	104	
Dyspnea	Negative	26	117	**0.012**
	Positive	4	3	
Crying^b^	Negative	21	112	**0.000**
	Positive	9	8	
Retching	Negative	13	43	0.447
	Positive	17	77	
Lymphadenomegaly	Negative	17	74	0.616
	Positive	13	46	
Pale mucous	Negative	12	65	0.165
	Positive	18	55	
Dehydration	Negative	7	25	0.765
	Positive	23	95	
Abnormal heart sound	Negative	30	118	0.477
	Positive	0	2	
Abnormal respiratory sound	Negative	28	108	0.575
	Positive	2	12	

The frequency of each variable has been compared between survivor and non-survivor groups.

^a^History of stress means any stress (especially weaning and transport) that the puppy has endured.

^b^Crying is defined as whining/whimping reported by the dog's owner and observed by the clinician during treatment. The bold values indicate statistically significant differences.

**Table 4:** Descriptive analysis of clinical numerical variables in studied dogs with CPV.

		***N***	**Mean**	**SD**	**SE**	**Min**	**Max**	***P*-value**
Temperature	None-survivor	29	38.8	0.9	0.2	36.2	40	0.587
	Survivor	120	38.9	0.7	0.1	36.5	40.7	
Anorexia	None-survivor	30	1.6	1.0	0.2	0	4	0.503
	Survivor	120	1.7	1.0	0.1	0	6	
Heart rate	None-survivor	30	140.4	30.9	5.6	84	209	0.076
	Survivor	120	130.7	25.6	2.3	70	194	
Respiratory rate	None-survivor	30	36.7	12.7	2.3	20	83	0.952
	Survivor	120	36.5	15.5	1.4	13	120	

Each variable has been compared between survivor and non-survivor groups.

We also evaluated hematology parameters first at admission and second ??? days after admission. None of the hematology parameters were different between survival and none-survival dogs in the first sampling (*Ps* > 0.05) (Supplementary Tables 1, 2), while in the second blood sampling, the mean RDW and mean platelet-to-lymphocyte ratio (PLR) were significantly higher in the none-survival group than in the survival group (66.9 vs. 14.3 and 793 vs. 273, respectively) (*Ps* < 0.05) (Table 5). In addition, the number of dogs with leukopenia and neutropenia in the non-survival group were significantly higher than in the survival group (*Ps* < 0.05) (Table 6). Although the hematology and cytopenia variables of the second sampling were found to be suitable prognostic factors, these variables were not included in the prognostic models because less than one third of the cases had a second sampling. Furthermore, our preference was to use only parameters that could be recorded at the time of admission, the other reason not including hematology and cytopenia at the second sampling in our models. Our analysis also showed that the serum levels of glucose, magnesium and PON enzyme were not statistically different in the two groups (*Ps* > 0.05) (Supplementary Table 3).

**Table 5:** Descriptive statistics and comparison of hematological parameters of the second sampling (postadmission) between two groups of survival and non-survival dogs infected with CPV.

		***N***	**Mean**	**SD**	**SE**	**Min**	**Max**	***P*-value**
WBC	None-survivor	7	7.3	6.2	2.4	0.4	13.1	0.226
	Survivor	40	10.9	7.0	1.1	0.4	30.9	
RBC	None-survivor	7	6.7	2.2	0.8	3.8	10.4	0.243
	Survivor	40	5.9	1.5	0.2	2.9	10.3	
HGB	None-survivor	7	13.8	4.9	1.8	7.3	22.1	0.312
	Survivor	40	12.3	3.5	0.5	5.7	21.3	
HCT	None-survivor	7	44.5	16.2	6.1	23.2	74.5	0.233
	Survivor	40	38.6	10.7	1.7	19.3	66.7	
PLT	None-survivor	7	424.4	253.3	95.7	154.0	957.0	0.088
	Survivor	39	304.2	138.9	22.2	108.0	807.0	
LY	None-survivor	7	1.3	1.6	0.6	0.0	4.6	0.12
	Survivor	39	2.5	2.1	0.3	0.1	12.0	
MO	None-survivor	7	0.1	0.1	0.0	0.0	0.3	0.234
	Survivor	39	0.3	0.4	0.1	0.0	1.7	
EO	None-survivor	7	0.2	0.4	0.2	0.0	1.2	0.54
	Survivor	39	0.3	0.4	0.1	0.0	1.4	
GR	None-survivor	7	5.6	5.3	2.0	0.1	12.3	0.568
	Survivor	39	7.6	5.9	0.9	0.4	26.0	
Band	None-survivor	7	0.1	0.3	0.1	0.0	0.7	0.1
	Survivor	39	0.5	0.5	0.1	0.0	1.7	
RDW	None-survivor	7	66.9	142.5	53.9	11.5	390.0	**0.018**
	Survivor	40	14.3	1.9	0.3	11.6	19.1	
NLR	None-survivor	7	8.1	14.3	5.4	0.3	40.0	0.295
	Survivor	39	5.3	6.8	1.1	0.2	30.4	
PLR	None-survivor	7	793.2	678.0	256.2	33.5	1914.0	**0.03**
	Survivor	39	273.1	543.4	87.0	28.6	3425.0	

The bold values indicate statistically significant differences.

**Table 6:** Frequency and statistical comparison of different parameters of cytopenia in the second sampling among two groups of survival and non-survival.

		**None-survivor**	**Survivor**	***P*-value**
Leukopenia	Negative	4	37	**0.003**
	Positive	3	2	
Neutropenia	Negative	4	36	**0.011**
	Positive	3	3	
Lymphopenia	Negative	3	28	0.143
	Positive	4	10	
Shift to left	Negative	3	20	0.679
	Positive	4	19	

The bold values indicate statistically significant differences.

### ML models to predict clinical outcome

As described in the Methods section, we went through three steps to arrive at a final model for robust prediction of clinical outcome in our patients. Primary and secondary models were trained and tested using 9 ML algorithms, while the final model was trained and tested using 10 algorithms. The performance of the models in two training and test groups was evaluated through three parameters: accuracy score, AUC score and AUC. We developed our models with and without SMOTE analysis (for dealing with imbalance dataset). In general, we obtained almost similar values for different metrics in both approaches, but two metrics including AUC score on the test dataset and AUC score on the training dataset were lower in models developed using SMOTE compared to models without SMOTE, while other metrics were remained unchanged. Hence, due to the higher performance metrics explained above, we preferred to consider the models developed without SMOTE analysis as the main models for further analysis (Tables 7–9; Supplementary Tables 4–6). Performance metrics for models developed using SMOTE analysis are presented in Supplementary Tables 4–6.

**Table 7:** Measuring the performance parameters of the primary model built with different algorithms in train and test groups.

	**Accuracy score (train)**	**Accuracy score (test)**	**AUC score (train)**	**AUC score (test)**	**AUC (test)**
LogisticRegression	0.94	0.81	0.96	0.72	0.51
SVC	0.95	0.79	0.97	0.67	0.47
GaussianProcessClassifier	1.00	0.52	1.00	0.65	0.50
DecisionTreeClassifier	1.00	0.71	1.00	0.53	0.52
RandomForestClassifier	0.99	0.79	0.99	0.65	0.62
AdaBoostClassifier	1.00	0.81	1.00	0.71	0.62
GaussianNB	0.83	0.77	0.75	0.65	0.61
LinearDiscriminantAnalysis	0.95	0.81	0.97	0.71	0.52
GradientBoostingClassifier	1.00	0.71	1.00	0.56	0.62

**Figure 4:**
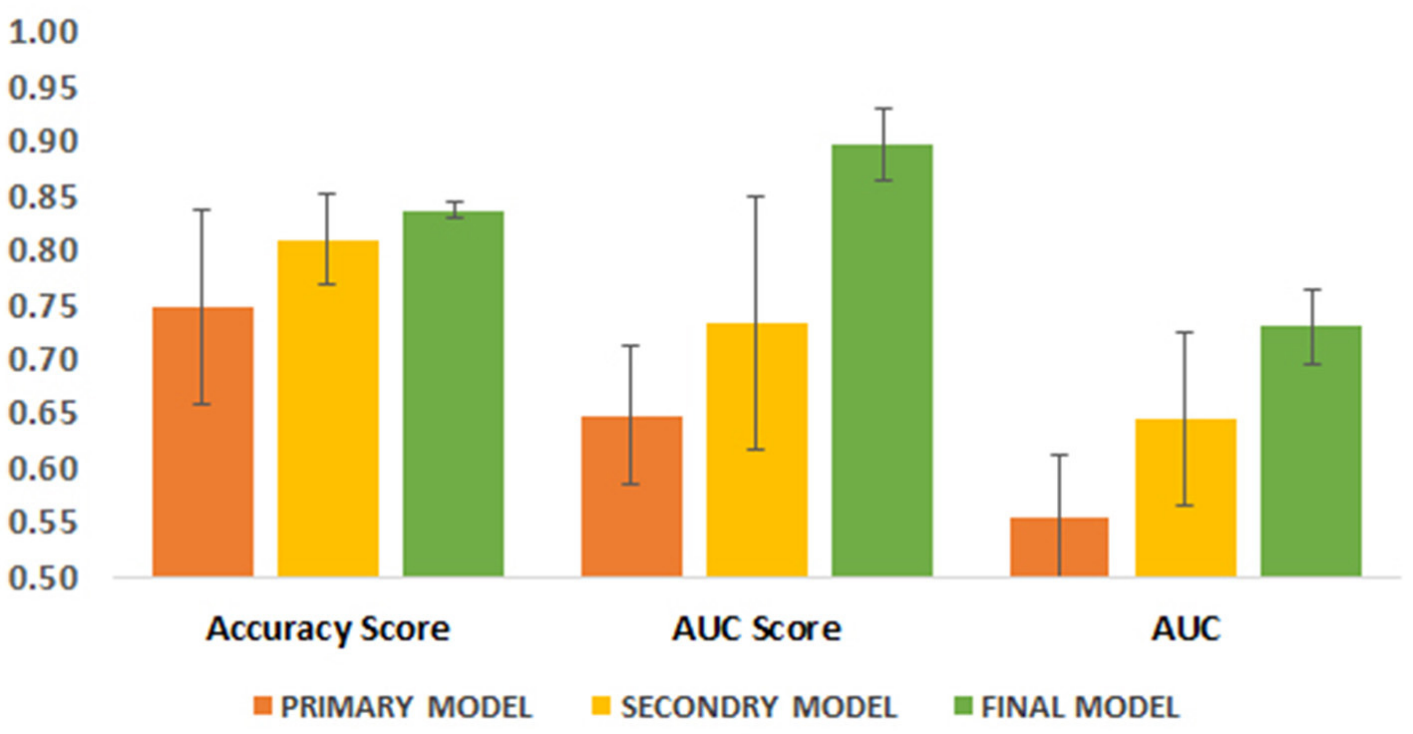
Comparison of three performance parameters between primary, secondary and final models in the test group. Each parameter is its average in different algorithms.

**Figure 5:**
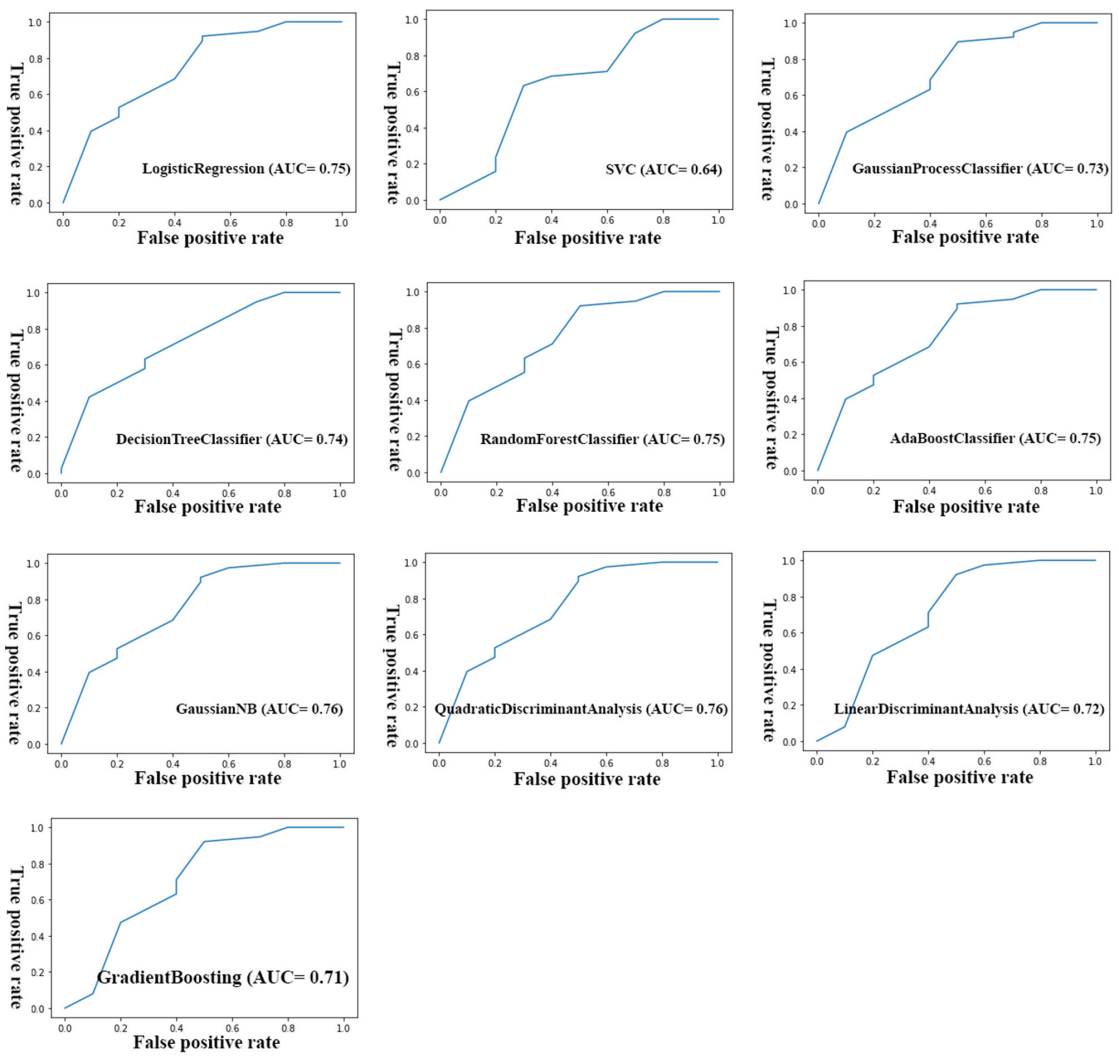
AUC plots of different final models built using different algorithms.

Although the primary models in the training group had high accuracy scores (mean = 0.96), these models performed poorly in the test group, so that the AUC scores (mean = 0.65) and AUCs (mean= 0.55) were poor and close to the random range (Table 7; Figure 4; Supplementary Figure 2). Due to the low performance and large number of variables in the primary model, after selecting seven important variables using logistic regression analysis, we developed the secondary model (Figure 2). In the secondary models, the average accuracy scores in the training and test groups were 0.88 and 0.81, respectively. Also, the average AUC score in the test group was good (0.73). However, mean AUCs (0.65), as the most important performance metric, were poor in secondary models (Table 8; Figure 4; Supplementary Figure 3). After conducting RFECV analysis to find robust features/(variables), the final model was developed using four variables (SIRS, deworming, vaccination and crying) (Figure 2). As shown, the performance of our final models improved significantly on the training and test datasets. The average accuracy score in the training and test groups was 0.82 and 0.84, respectively. In addition, the average of AUC scores and AUCs in the test group were excellent (0.90) and good (0.73), respectively (Table 9; Figures 4, 5). In summary, in an effort to improve the performance of the models, all performance parameters gradually increased from the initial model to the final model, of which the AUC score increased the most from the initial model to the final model (Figure 4).

**Table 8:** Measuring the performance parameters of the secondary model built with different algorithms in train and test groups.

	**Accuracy score (train)**	**Accuracy score (test)**	**AUC score (train)**	**AUC score (test)**	**AUC (test)**
LogisticRegression	0.81	0.83	0.91	0.80	0.72
GaussianProcessClassifier	0.92	0.77	0.95	0.61	0.48
DecisionTreeClassifier	1.00	0.73	1.00	0.55	0.53
RandomForestClassifier	0.98	0.79	0.99	0.66	0.68
AdaBoostClassifier	0.88	0.81	0.88	0.71	0.66
GaussianNB	0.77	0.88	0.62	0.86	0.72
QuadraticDiscriminantAnalysis	0.79	0.85	0.64	0.83	0.68
LinearDiscriminantAnalysis	0.83	0.83	0.82	0.91	0.68
GradientBoostingClassifier	0.96	0.79	0.98	0.68	0.66

**Table 9:** Measuring the performance parameters of the final model built with different algorithms in train and test groups.

	**Accuracy score (train)**	**Accuracy score (test)**	**AUC score (train)**	**AUC score (test)**	**AUC (test)**
LogisticRegression	0.81	0.83	0.91	0.91	0.75
SVC	0.82	0.83	0.79	0.91	0.64
GaussianProcessClassifier	0.81	0.83	0.91	0.91	0.73
DecisionTreeClassifier	0.83	0.83	0.82	0.91	0.74
RandomForestClassifier	0.83	0.83	0.82	0.91	0.75
AdaBoostClassifier	0.82	0.83	0.79	0.91	0.75
GaussianNB	0.79	0.85	0.64	0.83	0.76
QuadraticDiscriminantAnalysis	0.79	0.85	0.64	0.83	0.76
LinearDiscriminantAnalysis	0.82	0.83	0.79	0.91	0.72
GradientBoostingClassifier	0.82	0.83	0.79	0.91	0.71

We also evaluated our final model for the overfitting problem as an undesirable ML behavior using *k*-fold cross-validation analysis. In overfitting models, the developed model provides accurate predictions on the training dataset, while it performs poorly on the internal/external test dataset. Our analysis showed that our final models did not suffer from overfitting, as the average of accuracy scores for 10 independent analyzes were almost the same for all but two algorithms (i.e., *QuadraticDiscriminantAnalysis* and *LinearDiscriminantAnalysis*) in the training and test groups (Table 10). However, in the two mentioned algorithms, the average accuracy scores were not significantly different between the two datasets.

**Table 10:** K-fold test to check the presence of overfitting in the final model made using different algorithms (standard deviation was equal to zero in all analyses).

	**Average accuracy score (train)**	**Average accuracy score (test)**
LogisticRegression	0.83	0.82
SVC	0.83	0.82
GaussianProcessClassifier	0.82	0.82
DecisionTreeClassifier	0.83	0.82
RandomForestClassifier	0.83	0.82
AdaBoostClassifier	0.80	0.79
GaussianNB	0.82	0.79
QuadraticDiscriminantAnalysis	0.82	0.75
LinearDiscriminantAnalysis	0.82	0.75
GradientBoostingClassifier	0.83	0.82

### ML model to predict recovery time

We also developed a predictive model to estimate recovery time in studied dogs with CPV. Predictive models were trained and tested using four ML regression algorithms. In order to enter meaningful numerical and categorical variables in the model, we performed two primary statistical analyses. Among the categorical variables, only retching, foul smelling, housing, dehydration, and shift-to-left had a significant association with recovery time (Supplementary Table 7). Except body weight, all other numerical variables did not show significant correlation (*r* < 0.25) with recovery time (Supplementary Table 8). Body weight had a weak correlation with the dependent variable (*r* = 0.46). Hence, only retching, foul smelling, housing, shift-to-left and body weight were used to develop predictive models.

Recovery time in the studied dogs were 6 ± 1.8 days. The developed models predicted the recovery time in the test group with an average error rate of 2.05 days. Among the four models, the model developed using *LinearRegression* had the lowest error rate (RMSE = 1.86 days). Since the body weight variable had a weak correlation with the recovery time, this feature was removed in the next step and then the model was trained again. In the new model without body weight, the performance of the models improved slightly (mean RMASES = 1.88 days). Again, the developed *LinearRegression* model had the lowest error rate (RMSE = 1.81 days) (Table 11).

**Table 11:** Evaluating the performance of four regression algorithms for predicting recovery time.

	**MSES**	**RMSES**
LinearRegression	3.53	1.86
DecisionTreeRegressor	6.19	2.41
RandomForestRegressor	3.95	1.94
KNeighborsRegressor	4.24	2.02
LinearRegression	3.37	1.81
DecisionTreeRegressor	3.85	1.95
RandomForestRegressor	3.47	1.85
KNeighborsRegressor	3.81	1.94

The panel above the dotted line is the prediction using all the selected variables and the panel below the dotted line is the prediction using all the variables except body weight.

## Discussion

In this study, we developed models to predict clinical outcome and recovery time in dogs with CPV. Similar to our findings, in a study by Franzo et al. (18), ML models were developed using different algorithms to predict outcome in dogs with CPV infection, by using some hematology and serum biochemistry parameters, including antithrombin, serum aspartate aminotransferase, serum lipase, monocyte and lymphocyte count, in contrary to our study where we comprehensively combined various demographic, clinical and laboratory variables to develop the predictive models. As a key point in ML, we tried to build models with minimum number of variables so that these variables can be easily obtained/measured by users (here small animal clinicians). Clearly, models with a large number of variables or models with unusual and hard-to-measure variables (such as antithrombin) cannot be easily applied. Our variables in this model (SIRS, deworming, vaccination and crying) are readily available and can be effortlessly recorded by clinicians. In Franzo et al. model, *random forest* performed best, while in our final model, except for *SVC, GaussianNB*, and *QuadraticDiscriminantAnalysis*, all other 8 algorithms including *random forest* performed similarly in terms of performance criteria. Also, in the abovementioned study, no model was provided for estimating recovery time.

One of the major drawbacks of previous prognostic markers is that they usually work best 24–48 h after admission, whereas we need markers that would be useful at the time of admission. So that, we consider the variables that can be recorded at the time of admission. As shown in the results, although some hematology and cytopenia variables in the second sampling were significantly associated with clinical outcome, they were not included in the predictive models.

In our final model, the four variables of SIRS, deworming, vaccination, and crying were used to predict clinical outcome with acceptable performance. In agreement with our study, previous studies confirmed SIRS as a critical risk factor in non-surviving dogs with CPV, with dogs with SIRS having a higher mortality rate (5, 26, 27). Additionally, we found that vaccinated and dewormed pups had a lower risk of death compared to unvaccinated or untreated pups. It has been documented that vaccinated dogs had lower odds for developing CPV infection than unvaccinated dogs (4). It has been confirmed that the accumulation of parasites in the intestine can increase the severity of parvovirus enteritis in dogs (19, 28). Moreover, it was shown that sometimes a single anthelmintic treatment could be associated with an increased risk of parvovirus infection (4). As a surprising finding in our study, we found that crying was significantly associated with the risk of death in CPV-infected dogs, with crying occurring in 30% of non-surviving dogs compared to only 6.6% of surviving dogs (Table 3). To the best of our knowledge, this is the first study to introduce crying as a potential prognostic factor to predict clinical outcome in CPV enteritis.

Consistent with previous studies, reporting that parvovirus infection usually occurs in pups < 6 months of age (4, 5, 20), the mean age of our cases was 4.6 months. Furthermore, we similarly found no relationship between breed and risk of CPV enteritis (5, 29). Some studies reported breed predisposition for Doberman Pinscher and Rottweiler breeds (4, 20). Because these breeds were present in very small numbers in our study, we could not find such a significant association. In our project, we found that hematological and cytopenic variables at the time of admission did not differ between surviving and none-surviving dogs, while some of these variables (leukopenia, neutropenia, RDW and PLR) in the second sampling after admission were significantly different between the two groups. Likewise, the occurrence of leukopenia, neutropenia, and lymphopenia 24 and 48 h after admission has been reported as risk factors in none-survivor dogs with CPV enteritis (6, 20). In addition, some studies showed that initial leukopenia, neutropenia, or lymphopenia decreased the chance of survival (19, 30). Although variables such as dyspnea, PLR, NLR, and RDW are important parameters in CPV, they were not considered significant based on statistical and machine learning tests such as logistic regression analysis and other algorithms and therefore were not included in the model.

Our study also had limitations that reduced the quality of the developed models. First, we had a relatively small sample size (*n* = 156), which may have negatively affected the performance of the models. Clearly, with a larger population of dogs with CPV, we can achieve more powerful models with excellent AUCs. However, by using three serial screenings, we could achieve final models with strong AUC values, which could be generalized in highly populated models. The second problem was the weakness of recording information due to the lack of cooperation of clinicians or animal owners. Hence, we had to remove some cases with high missing variables. Despite all these issues, the models used in this study can be developed using a larger population of dogs and by applying more extensive data (variables) to achieve more efficient ML models for survival prediction.

## Data Availability

The original contributions presented in the study are included in the article/Supplementary material, further inquiries can be directed to the corresponding author.

## Supplementary material

Supplementary Figure 1 The workflow for removing non-informative variables.

Supplementary Figure 2 AUC plots of different primary models built using different algorithms.

Supplementary Figure 3 AUC plots of different final secondary built using different algorithms.

Supplementary Table 1 Descriptive statistics and comparison of hematological parameters of the first sampling between two groups of survival and non-survival dogs infected with CPV.

Supplementary Table 2 Frequency and statistical comparison of different parameters of cytopenia in the first sampling among two groups of survival and non-survival.

Supplementary Table 3 Descriptive statistics and comparison of three biochemical parameters (Mg, glucose and paraoxonase enzyme) between two surviving and non-surviving groups.

Supplementary Table 4 Measuring the performance parameters of the primary model built using SMOTE analysis and different algorithms in train and test groups.

Supplementary Table 5 Measuring the performance parameters of the secondary model built using SMOTE analysis and different algorithms in train and test groups.

Supplementary Table 6 Measuring the performance parameters of the final model built using SMOTE analysis and different algorithms in train and test groups.

Supplementary Table 7 Correlation between categorical variables and recovery time. Values in this table are P values. Significant variables are in bold.

Supplementary Table 8 Correlation coefficient between numerical variables and recovery time. Only body weight had a weak correlation with recovery time.
