# Supplementary figures and images for: Development of machine learning models to predict clinical outcome and recovery time in dogs with parvovirus enteritis
Source: Front Vet Sci. 2025 Apr 15;12:1555714. doi: 10.3389/fvets.2025.1555714 (PMC12037471; doi:10.3389/fvets.2025.1555714)

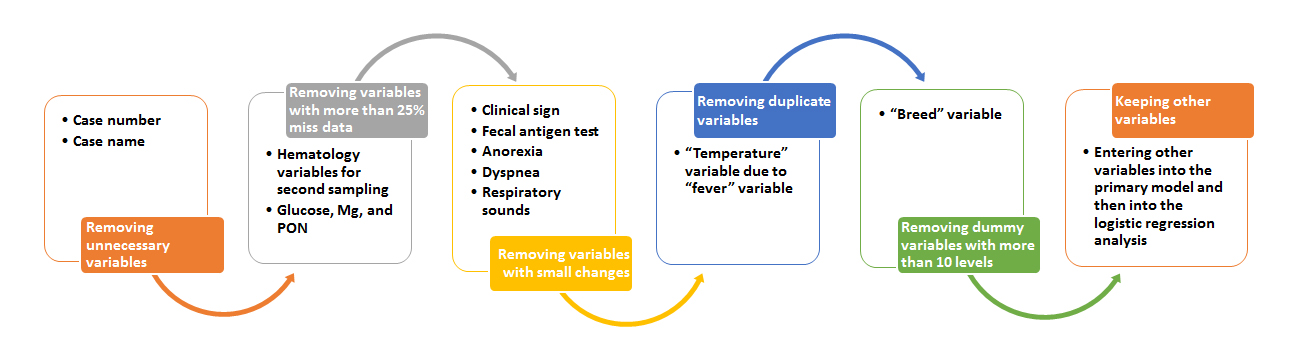

Supplement: Supplementary Figure 1 — The workflow for removing non-informative variables. [file Image_1.jpeg]

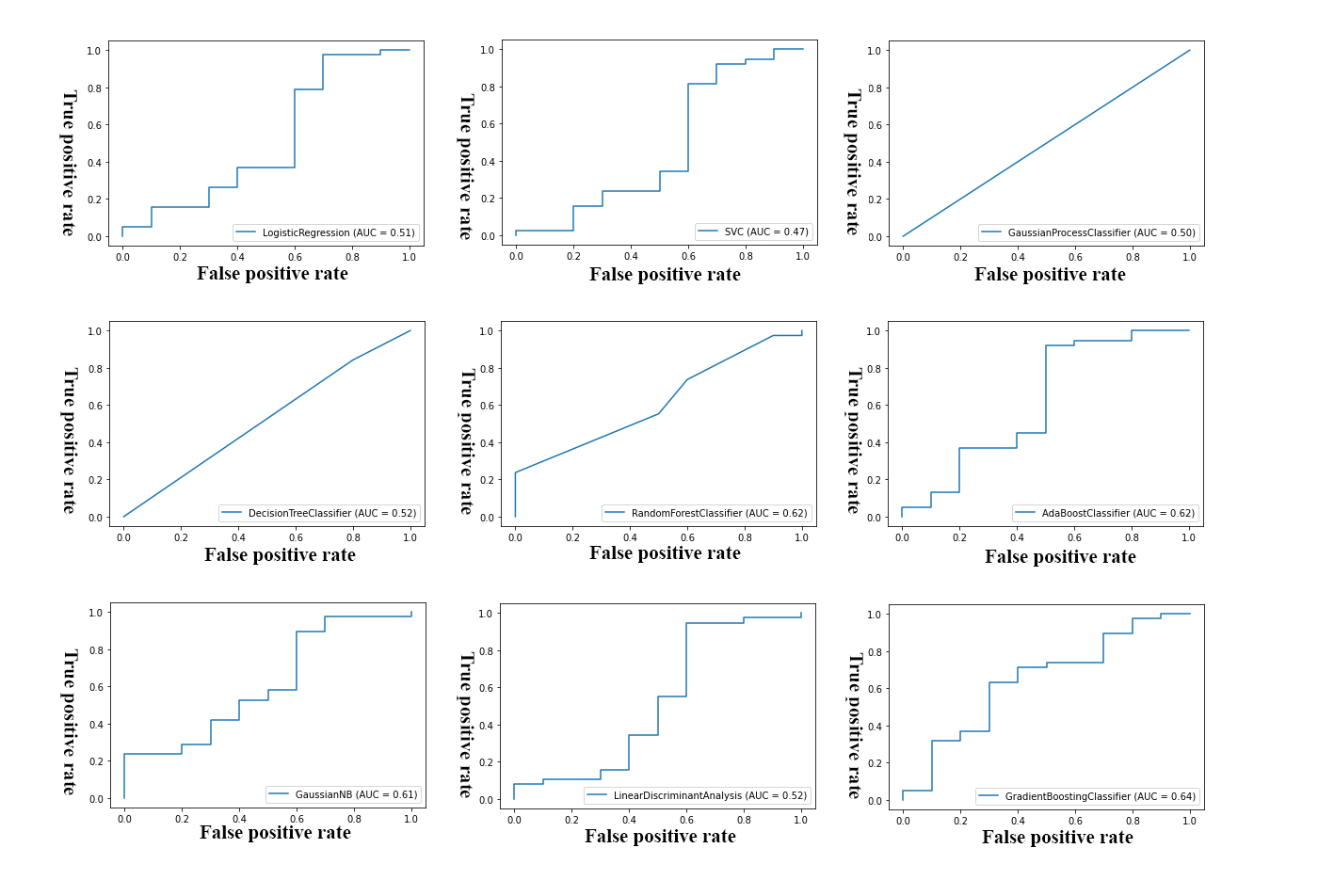

Supplement: Supplementary Figure 2 — AUC plots of different primary models built using different algorithms. [file Image_2.jpeg]

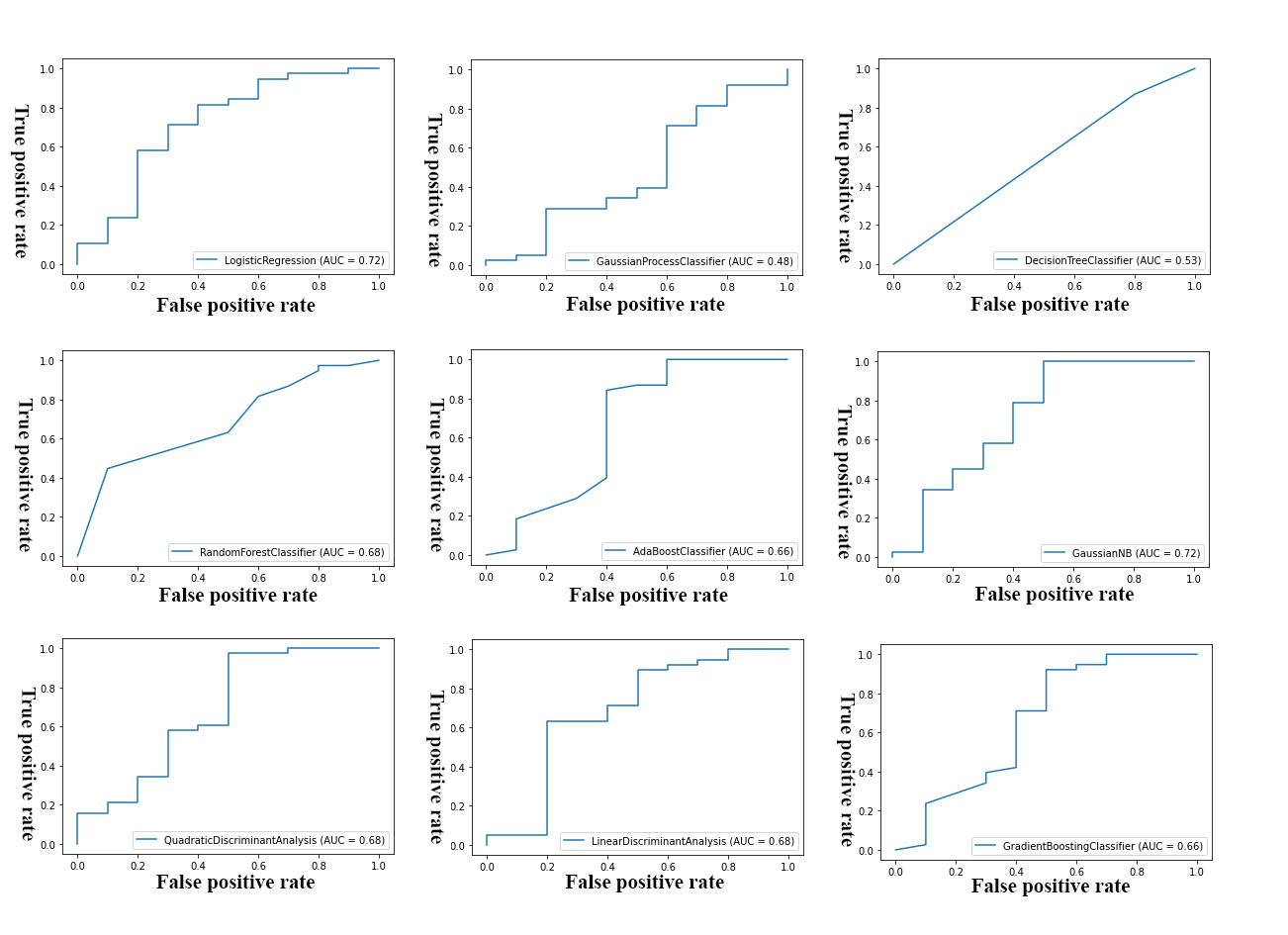

Supplement: Supplementary Figure 3 — AUC plots of different final secondary built using different algorithms. [file Image_3.jpeg]
