# Supplementary material for: Development of machine learning models to predict clinical outcome and recovery time in dogs with parvovirus enteritis
Source: Front Vet Sci. 2025 Apr 15;12:1555714. doi: 10.3389/fvets.2025.1555714 (PMC12037471; doi:10.3389/fvets.2025.1555714)
Supplement: Supplementary Table 1 — Descriptive statistics and comparison of hematological parameters of the first sampling between two groups of survival and non-survival dogs infected with CPV. [file Table_1.docx]

| **Supplementary Table 1:**Descriptive statistics and comparison of hematological parameters of the first sampling between two groups of survival and non-survival dogs infected with CPV | | | | | | | | |
| --- | --- | --- | --- | --- | --- | --- | --- | --- |
|  |  | *N* | *Mean* | *SD* | *SE* | *Min* | *Max* | *P-value* |
| **WBC** | None-survivor | 30.0 | 10.8 | 7.5 | 1.4 | 1.2 | 30.2 | 0.383 |
|  | Survivor | 120.0 | 10.6 | 5.2 | 0.5 | 1.0 | 24.8 |  |
| **RBC** | None-survivor | 30.0 | 5.4 | 1.9 | 0.4 | 1.1 | 9.2 | 0.174 |
|  | Survivor | 120.0 | 5.8 | 1.3 | 0.1 | 1.3 | 10.3 |  |
| **HGB** | None-survivor | 30.0 | 11.3 | 4.4 | 0.8 | 1.5 | 20.8 | 0.119 |
|  | Survivor | 120.0 | 12.2 | 3.1 | 0.3 | 3.0 | 21.7 |  |
| **HCT** | None-survivor | 30.0 | 35.5 | 13.3 | 2.4 | 5.2 | 64.6 | 0.159 |
|  | Survivor | 120.0 | 37.7 | 9.3 | 0.9 | 9.2 | 68.9 |  |
| **PLT** | None-survivor | 30.0 | 280.4 | 186.8 | 34.1 | 33.0 | 931.0 | 0.418 |
|  | Survivor | 120.0 | 282.4 | 144.4 | 13.2 | 40.0 | 967.0 |  |
| **LY** | None-survivor | 26.0 | 2.1 | 2.0 | 0.4 | 0.1 | 10.0 | 0.137 |
|  | Survivor | 116.0 | 1.7 | 1.3 | 0.1 | 0.0 | 6.8 |  |
| **MO** | None-survivor | 26.0 | 0.5 | 0.5 | 0.1 | 0.0 | 1.6 | 0.531 |
|  | Survivor | 116.0 | 0.9 | 1.9 | 0.2 | 0.0 | 14.3 |  |
| **EO** | None-survivor | 26.0 | 0.1 | 0.1 | 0.0 | 0.0 | 0.6 | 0.063 |
|  | Survivor | 116.0 | 0.2 | 0.3 | 0.0 | 0.0 | 1.5 |  |
| **GR** | None-survivor | 26.0 | 9.1 | 5.9 | 1.2 | 0.5 | 24.5 | 0.148 |
|  | Survivor | 116.0 | 7.8 | 4.7 | 0.4 | 0.3 | 22.4 |  |
| **Band** | None-survivor | 26.0 | 0.4 | 0.4 | 0.1 | 0.0 | 1.7 | 0.8 |
|  | Survivor | 116.0 | 0.6 | 1.8 | 0.2 | 0.0 | 18.1 |  |
| **RDW** | None-survivor | 27.0 | 15.0 | 3.4 | 0.6 | 11.4 | 24.5 | 0.346 |
|  | Survivor | 120.0 | 14.2 | 3.1 | 0.3 | 0.4 | 29.8 |  |
| **NLR** | None-survivor | 26.0 | 11.0 | 20.8 | 4.1 | 0.2 | 105.3 | 0.253 |
|  | Survivor | 115.0 | 8.5 | 10.0 | 0.9 | 0.2 | 49.2 |  |
| **PLR** | None-survivor | 26.0 | 252.1 | 257.6 | 50.5 | 20.2 | 997.8 | 0.805 |
|  | Survivor | 115.0 | 274.5 | 268.4 | 25.0 | 1.0 | 1934.0 |  |

| **Supplementary Table 2:** Frequency and statistical comparison of different parameters of cytopenia in the first sampling among two groups of survival and non-survival | | | | |
| --- | --- | --- | --- | --- |
|  |  | *None-survivor* | *Survivor* | *P value* |
| **Leukopenia** | Negative | 22 | 98 | 0.419 |
|  | Positive | 8 | 22 |  |
|  |  |  |  |  |
| **Neutropenia** | Negative | 22 | 98 | 0.42 |
|  | Positive | 8 | 22 |  |
|  |  |  |  |  |
| **Lymphopenia** | Negative | 13 | 59 | 0.788 |
|  | Positive | 17 | 61 |  |
|  |  |  |  |  |
| **Shift to left** | Negative | 9 | 55 | 0.117 |
|  | Positive | 21 | 65 |  |

| **Supplementary Table 3:** Descriptive statistics and comparison of three biochemical parameters (Mg, glucose and paraoxonase enzyme) between two surviving and non-surviving groups | | | | | | | | |  |
| --- | --- | --- | --- | --- | --- | --- | --- | --- | --- |
|  |  | *N* | *Mean* | *SD* | *SE* | *Min* | *Max* | *P-value* | |
| **Mg** | None-survivor | 22 | 1.5 | 0.6 | 0.1 | 0.7 | 3.2 | 0.3 | |
|  | Survivor | 72 | 1.7 | 0.6 | 0.1 | 0.3 | 2.8 |  | |
| **Glucose** | None-survivor | 22 | 102.3 | 43.4 | 9.3 | 54 | 230 | 0.1 | |
|  | Survivor | 72 | 114.7 | 30.6 | 3.6 | 51 | 217 |  | |
| **PON** | None-survivor | 20 | 197.1 | 72.9 | 16.3 | 69.73 | 322.83 | 0.5 | |
|  | Survivor | 71 | 214.4 | 108.5 | 12.9 | 50.1 | 634.81 |  | |

| **Supplementary Table 4**: Measuring the performance parameters of the primary model built using SMOTE analysis and different algorithms in train and test groups. | | | | | | |
| --- | --- | --- | --- | --- | --- | --- |
|  | **Accuracy score (train)** | **Accuracy score**  **(test)** | **AUC score**  **(train)** | **AUC score**  **(test)** | **AUC**  **(train)** | **AUC**  **(test)** |
| LogisticRegression | 0.95 | 0.79 | 0.97 | 0.66 | 0.93 | 0.48 |
| SVC | 0.95 | 0.73 | 0.97 | 0.57 | 0.91 | 0.45 |
| GaussianProcessClassifier | 1 | 0.52 | 1 | 0.65 | 1 | 0.5 |
| DecisionTreeClassifier | 1 | 0.62 | 1 | 0.5 | 1 | 0.51 |
| RandomForestClassifier | 0.99 | 0.79 | 0.99 | 0.67 | 1 | 0.57 |
| AdaBoostClassifier | 1 | 0.75 | 1 | 0.62 | 1 | 0.55 |
| GaussianNB | 0.8 | 0.73 | 0.74 | 0.58 | 0.88 | 0.6 |
| LinearDiscriminantAnalysis | 0.95 | 0.79 | 0.97 | 0.67 | 0.96 | 0.5 |
| GradientBoostingClassifier | 1 | 0.73 | 1 | 0.57 | 1 | 0.55 |

|  | **Supplementary Table 5**: Measuring the performance parameters of the secondary model built using SMOTE analysis and different algorithms in train and test groups. | | | | | | | |
| --- | --- | --- | --- | --- | --- | --- | --- | --- |
|  | | **Accuracy score (train)** | **Accuracy score**  **(test)** | **AUC score**  **(train)** | **AUC score**  **(test)** | **AUC**  **(train)** | **AUC**  **(test)** |  |
| LogisticRegression | | 0.79 | 0.83 | 0.89 | 0.79 | 0.74 | 0.73 |  |
| GaussianProcessClassifier | | 0.98 | 0.77 | 0.97 | 0.63 | 0.99 | 0.48 |  |
| DecisionTreeClassifier | | 1 | 0.75 | 1 | 0.65 | 1 | 0.69 |  |
| RandomForestClassifier | | 0.98 | 0.83 | 0.97 | 0.79 | 1 | 0.56 |  |
| AdaBoostClassifier | | 0.86 | 0.83 | 0.92 | 0.76 | 0.95 | 0.71 |  |
| GaussianNB | | 0.77 | 0.85 | 0.67 | 0.79 | 0.75 | 0.76 |  |
| QuadraticDiscriminantAnalysis | | 0.76 | 0.83 | 0.65 | 0.76 | 0.83 | 0.67 |  |
| LinearDiscriminantAnalysis | | 0.82 | 0.79 | 0.84 | 0.66 | 0.75 | 0.73 |  |
| GradientBoostingClassifier | | 0.99 | 0.77 | 0.99 | 0.64 | 1 | 0.7 |  |

|  | **Supplementary Table 6**: Measuring the performance parameters of the final model built using SMOTE analysis and different algorithms in train and test groups. | | | | | | |
| --- | --- | --- | --- | --- | --- | --- | --- |
|  | | **Accuracy score (train)** | **Accuracy score**  **(test)** | **AUC score**  **(train)** | **AUC score**  **(test)** | **AUC**  **(train)** | **AUC**  **(test)** |
| LogisticRegression | | 0.78 | 0.83 | 0.89 | 0.91 | 0.81 | 0.75 |
| SVC | | 0.81 | 0.83 | 0.82 | 0.91 | 0.81 | 0.75 |
| GaussianProcessClassifier | | 0.78 | 0.83 | 0.89 | 0.91 | 0.82 | 0.74 |
| DecisionTreeClassifier | | 0.81 | 0.81 | 0.73 | 0.72 | 0.84 | 0.75 |
| RandomForestClassifier | | 0.81 | 0.81 | 0.73 | 0.72 | 0.83 | 0.75 |
| AdaBoostClassifier | | 0.79 | 0.83 | 0.77 | 0.91 | 0.81 | 0.75 |
| GaussianNB | | 0.77 | 0.85 | 0.65 | 0.83 | 0.80 | 0.76 |
| QuadraticDiscriminantAnalysis | | 0.77 | 0.85 | 0.65 | 0.83 | 0.80 | 0.76 |
| LinearDiscriminantAnalysis | | 0.81 | 0.83 | 0.82 | 0.91 | 0.80 | 0.72 |
| GradientBoostingClassifier | | 0.79 | 0.83 | 0.77 | 0.91 | 0.81 | 0.71 |

| Supplementary Table 7: Correlation between categorical variables and recovery time. Values in this table are *P* values. Significant variables are in bold. | | | | |
| --- | --- | --- | --- | --- |
| Gender | 0.127 |  | Dyspnea | 0.502 |
| Housing | **0.012** |  | Crying | 0.933 |
| Vaccination | 0.189 |  | **Retching** | **0.049** |
| Deworming | 0.158 |  | Lymphadenomegaly | 0.659 |
| Vaccination of mother | 0.522 |  | Pale mucous | 0.137 |
| History of stress | 0.960 |  | **Dehydration** | **0.035** |
| Fever | 0.167 |  | Abnormal respiratory sound | 0.540 |
| Anorexia | 0.844 |  | Recovery | 0.392 |
| Lethargia | 0.094 |  | Leukopenia | 0.642 |
| SIRS | 0.081 |  | Neutropenia | 0.201 |
| Vomiting | 0.066 |  | Lymphooenia | 0.184 |
| Foul smelling | **0.009** |  | **Shift to left** | **0.030** |

| **Supplementary Table 8:** Correlation coefficient between numerical variables and recovery time. Only body weight had a weak correlation with recovery time. | |
| --- | --- |
| LY | -0.132 |
| WBC | -0.096 |
| RDWCV | -0.072 |
| PLT | -0.064 |
| NLR | -0.057 |
| GR | -0.057 |
| MO | -0.007 |
| Band | 0.000 |
| HR | 0.000 |
| PLR | 0.009 |
| RR | 0.024 |
| Time_of_anorexia | 0.034 |
| RBC | 0.135 |
| EO | 0.149 |
| HGB | 0.150 |
| Age | 0.165 |
| HCT | 0.166 |
| **BW** | 0.461 |
